# The use of game modes to promote engagement and social involvement in multi-user serious games: a within-person randomized trial with stroke survivors

**DOI:** 10.1186/s12984-021-00853-z

**Published:** 2021-04-14

**Authors:** Fábio Pereira, Sergi Bermúdez i Badia, Carolina Jorge, Mónica S. Cameirão

**Affiliations:** 1 Faculdade de Ciências Exatas e da Engenharia, Universidade da Madeira, Campus Universitário da Penteada, 9020-105 Funchal, Portugal; 2 Madeira Interactive Technologies Institute, Universidade da Madeira, Pólo Científico e Tecnológico da Madeira, Caminho da Penteada, 9020-105 Funchal, Portugal; 3 NOVA Laboratory for Computer Science and Informatics, Caparica, Portugal

**Keywords:** Game mode, Serious games, Engagement, Social involvement, Rehabilitation, Stroke

## Abstract

**Background:**

Serious games are promising for stroke rehabilitation, with studies showing a positive impact on reducing motor and cognitive deficits. However, most of the evidence is in the context of single-user rehabilitation, and little is known concerning the impact in multi-user settings. This study evaluates the impact that different game modes can have on engagement and social involvement during a two-user game. Specifically, we want to understand the benefits of game modalities based on competition, co-activation, and collaboration and analyze the influence of different motor and cognitive deficits and personality traits.

**Methods:**

We developed a two-player setup—using tangible objects and a large screen interactive table—for upper limb rehabilitation purposes. We implemented a game that, while keeping the same basic mechanics, can be played in the three different modes (Competitive, Co-active, and Collaborative). We ran a within-person randomized study with 21 stroke survivors that were paired and played the game in its three versions. We used the Game Experience Questionnaire—Core Module to assess engagement and the Social Presence Module to assess Social Involvement. For personality, motor, and cognitive function, users answered the International Personality Item Pool (short version), Fugl-Meyer Assessment—Upper Extremity, Modified Ashworth Scale, and Montreal Cognitive Assessment, respectively.

**Results:**

The Collaborative mode promoted significantly more Behavioral Involvement. The Competitive mode promoted more Flow and Challenge than the Co-active mode with participants with better cognitive performance, with low extraversion, or with higher motor skills. Participants with higher cognitive deficits reported more Competence with the Co-active mode.

**Conclusions:**

Our results indicate that, for multi-user motor rehabilitation settings, the collaborative mode is the more appropriate gaming approach to promote social involvement, showing a high potential for increasing adherence and effectiveness of therapy. Additionally, we show that a player's motor and cognitive ability and personality should be considered when designing personalized tasks for multiplayer settings.

## Introduction

The use of novel technologies for neurorehabilitation has increased during the last years, leading to new rehabilitation methods with multifold benefits [1]. Depending on the technology, we can benefit from personalization to individual patients' specific needs, the ability to measure with objectivity, or provide visual, haptic, or auditory real-time feedback [2]. Additional benefits emerge with the use of Virtual Reality (VR), which can provide enriched environments for stroke survivors to engage in problem-solving challenges and therefore develop new skills [3]. VR combined with serious gaming offers attractive rehabilitation options because motor learning principles underlying neuroplasticity, such as practice, augmented feedback, motivation, and observational learning [4], are inherent features of VR systems [5]. Moreover, VR allows us to define goal-oriented tasks and promotes more task repetitions than conventional therapy, which have been shown to be essential for neurological rehabilitation [6, 7]. Finally, the engagement with VR based approaches has been shown to lead to high treatment adherence, with patients reporting that it is more interesting and enjoyable than standard care [1, 8, 9].

Despite the many benefits of technology-mediated rehabilitation approaches, other aspects such as the environment, changes in assistive devices, individual preferences, and interaction with peers can modulate the delivered experience and its impact on the users [2, 3]. For instance, multi-user user technology-mediated rehabilitation approaches have been shown to influence rehabilitation outcomes, highlighting the potential positive effects of social interaction in rehabilitation settings [10]–[12]. However, features like the way players interact between themselves to achieve success in a task or game, i.e., the interaction mode (competitive, cooperative, or collaborative), can influence the social impact and engagement experienced by the users [10, 11]. In fact, Baur et al. identified nine studies where different multiplayer modes had a different effect on game experience [12]. In general, competitive games have been shown to lead to higher enjoyment [13]–[15]. Specifically, competitive game modes seem to be related to higher physical effort and usually require more skills, at least more than the opponent, to produce performance satisfaction when compared to modalities that require collaboration or cooperation [16–19]. However, there is no consensus on this matter, as there is literature suggesting that cooperative modes lead to greater efforts than their competitive counterparts [20]. Collaborative modes have been less addressed in the literature, and therefore the evidence on their specific impact is still scarce [12, 21–24]. Also, to our best knowledge, only two recent studies compared the three game modes (competitive, cooperative, and collaborative) [10, 11], with most studies comparing cooperative with competitive modes [12].

When reviewing the specific impact of cooperative and collaborative modes, we found that Roschelle et al. define collaboration as "to work together," which requires coordination of efforts to solve a problem and establish a synergic relationship [25] and cooperation as "to operate together," which means to divide the work among different operators, each one being responsible for a portion of a problem. In contrast, a recent review study defines cooperation as "playing in the same team with different roles according to own individual skills; thus, a role being either "supported" or "supportive"” [12]. These authors go further and suggest that in a substantial number of studies, modes termed as cooperative should have been termed as co-active instead because the individual player can solve the task by itself without depending on the co-player [12]. In our here presented study, we adopted this renaming of cooperative to co-active mode as we believe that the therapeutic impact of a game can be very different if players have the same or different tasks on a multi-user game.

We developed a multi-user interactive table with a custom-made serious game intended to enhance the social impact and improve self-efficacy during motor rehabilitation of stroke survivors. In this study, we aim to understand what the impact on engagement and social involvement of stroke survivors is of having the game presented in three different modes, namely, Competitive, Co-active, and Collaborative. For this purpose, we recruited a sample of stroke survivors who were paired to play a game in those three game modes. We investigated competitive, cooperative, and collaborative modes in a previous study with community-dwelling older adults using a different experimental setup (a vertical screen with indirect interaction with VR), identifying some positive effects in collaborative interaction [10]. However, it is not sure to what extent the previously observed results generalize to a stroke population with motor deficits, a different interaction modality (direct interaction with VR), and different technology and interfaces. We hypothesize that engagement will be higher in the Competitive mode than Co-active and Collaborative modes, as the literature suggests that competitive modes are usually more motivating [26]. Also, we expected social involvement to be higher in the Collaborative game mode when compared to Competitive and Co-active modes because this specific mode requires dependence on the co-player to reach the goal. Finally, we want to understand how the results are affected by different levels of motor and cognitive function, spasticity, and personality.

## Methods

### Experimental setup

**Fig. 1:**
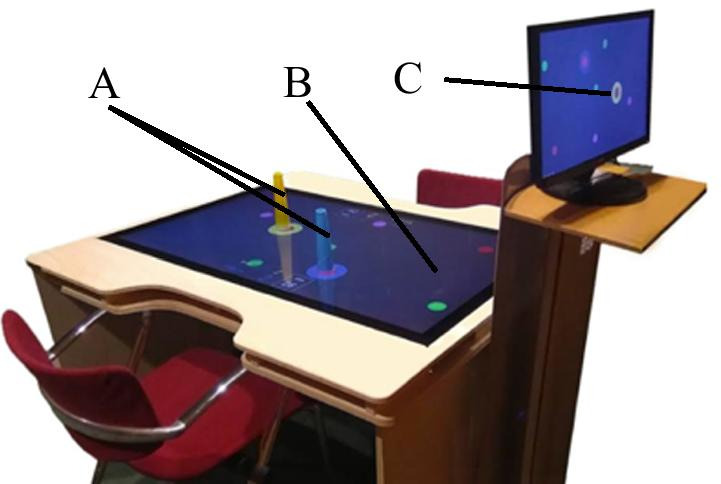
Experimental Setup: Handles (**a**), infrared touch sensitive layer (**b**), and auxiliary screen for the researcher (**c**)

The interactive table setup consisted of a PC (OS: Windows 10, CPU: Intel Coffee Lake Core I7-8700K 3.7 GHz 12 MB, RAM: 2x 8GB DDR4 2400 Mhz, Graphics: Gigabyte Nvidia GTX 1070 Ti Gaming 8G GDDR5), a 55" LED TV screen and a 55" infrared multitouch sensitive layer (latency: < 15 ms panning, < 25 ms touchdown, reporting rate 100 Hz), plus an auxiliary screen for the researcher (Fig. 1). Users were seated facing each other, with arms resting on the table and hands on the touch-sensitive screen. Users used a plastic cone (5 cm base diameter, 17.5 cm height, and 3.8 cm top diameter)—later referred to as the handle—mounted on a soft conductive base to interact with the game. This base reduced friction and facilitated detection over the touchscreen. This object is commonly used for rehabilitation purposes as it eases basic prehension (global prehension) and keeps it uniform through all participants. We used chairs with adjustable height and a structure underneath the table to rest the feet to guarantee a proper posture while seated.

### Task

**Fig. 2:**
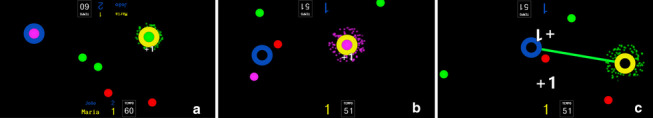
Competitive (**a**), Co-active (**b**) and Collaborative (**c**) game modes, from top to bottom, respectively. The rings (yellow and blue) are used to catch the balls (inside they have the color of the last ball caught) and represent the color of the player. Each mode shows a score and time left to end the round

The task was a two-player game whose primary objective was to catch balls that appeared in random positions, moving straight to make their movement predictable and easier for users to anticipate. It was designed to have straightforward mechanics to guarantee quick learning. Additionally, its simplicity allowed us to adapt it to the three different game modes with minor game mechanics changes. We opted to have the balls move on a straight line to make their movement predictable and allow easier planning. Each user controlled a virtual ring on the screen by moving the handle over the surface. We implemented three different versions of the task, each corresponding to a different game mode: Competitive, Co-active, and Collaborative (Fig. 2). The different game modes relied on the same basic mechanics. In the Competitive mode (Fig. 2a), each participant had to catch the maximum number of balls, which accumulated to his/her score. The participant who scored more points (balls caught) would win the round. In the Co-active (Fig. 2b) game mode, participants had to play as a team and catch balls, but points would accumulate to a single combined team score. Finally, in the Collaborative (Fig. 2c) game mode, participants also played as a team but only scored if both players consecutively caught two balls of the same color. These game modes were chosen because Competitive is a game mode that allows participants to engage in a task to be superior to the opponent. In the Collaborative and Co-active modes, the perspective is very different; they have to work as a team. However, in the Collaborative mode, they are dependent on each other to reach the goal, whereas, in the Co-active mode, they do not depend on each other.

### Sample and recruitment

A convenience sample of stroke survivors was recruited in health units of the regional health service (SESARAM) in Madeira Island, Portugal. The inclusion criteria were to have suffered a stroke and sustaining upper limb motor deficits. The exclusion criteria were to be unable to hold the handle used for the interaction with the table, to have any type of aphasia diagnosed, not having the ability to read, and hemispatial neglect, assessed through Bells Test [27].

Two hundred and seventeen potential participants were approached. Thirteen refused to participate, and 184 were excluded due to exclusion criteria. The reason for this high exclusion percentage was the way of contact, i.e., personally or by a phone call. One hundred and two potential participants were contacted by a phone call, as the local health services gave access to a list of recent stroke survivors and the respective phone number. Sixty-eight of them reported not to have motor deficits. Fifty-three potential participants were excluded after a first approach because they presented minor motor difficulties (18), hemiplegia (18), cognitive deficits (7), aphasia (6), and 4 did not know how to read and write. Additionally, the first exclusion criteria (must be able to hold the handle) also implied the users to hold the handle (cone) with enough control to keep their forearm in a neutral position, allowing them to interact with the touch display.

One participant was excluded after data collection as he could not properly answer questionnaires due to having unreported aphasia. Twenty participants (12 males) with a mean age (with standard deviation) of 60.4 ± 8.2 years (range 31–71 years) concluded the study and were included in data analysis (Table 1). Five participants reported having previous experience with video games. To obtain a profile of the participants, we used: (1) A brief questionnaire for demographic information; (2) The Montreal Cognitive Assessment (MoCA) for cognitive screening [28]; (3) The Fugl-Meyer Assessment for Upper Extremity (FMA-UE) [29]; The Modified Ashworth Scale [30]; and The Mini-IPIP (Short version of International Personality Item Pool) validated for Portuguese population [31]. Although Mini-IPIP measures the Big Five personality factors (Openness/Intellect or Imagination, Conscientiousness, Extraversion, Agreeableness, and neuroticism), we only considered the extraversion factor as it is the one that we believe clearly relates to engagement and social involvement. The study was approved by the Ethics Commission of SESARAM (number 24/2018), and all participants provided written informed consent.

**Table 1:** Participants' profile

Pairs	Participant	Gender (M/F)	Age	TIME SINce stroke (months)	MoCA (0-30)	FMA-UE (0-66)	Ashworth (0–3)	Extraversion (5–20)
1	1	M	61	42	22	58	1	14
	2	M	53	23	23	58	0	16
2	3	M	60	74	30	47	1+	14
	4	F	71	44	20	55	0	17
3	5	F	31	54	29	17	2	10
	6	F	67	37	23	33	1+	12
4	7	M	64	8	27	47	1	9
	8	F	64	33	14	52	0	18
5	9	F	63	8	19	54	0	19
	10	F	59	51	25	56	1	11
6	11	F	61	178	28	24	3	11
	12	M	77	13	15	26	3	15
7	13	M	63	16	20	54	1	15
	14	M	65	46	12	53	0	12
8	15	M	62	4	21	31	1	16
	16	M	61	8	25	26	1	12
9	17	F	57	2	22	51	0	12
	18	M	64	1	16	56	0	18
10	19	M	55	5	12	29	1	11
	20	M	57	3	18	29	1	12

### Outcome measures

The Game Experience Questionnaire (GEQ)—Core Module [32] and the GEQ—Social Presence Module [33] were chosen to measure engagement and social involvement, respectively. The Core Module measures the players' thoughts and feelings through 7 components (Competence, Sensory and Imaginative Immersion, Flow, Tension/Annoyance, Challenge, Negative affect, and Positive affect) in a total of 33 items [32]. The Social Presence Module has three components (Psychological Involvement—Empathy, Psychological Involvement—Negative Feelings and Behavioral Involvement) and 17 items. In both questionnaires, the items are rated as "0" (Not at all), "1" (Slightly), "2" (Moderately, "3" (Fairly), or "4" (Extremely). These questionnaires are typically filled-in by the user, but because of the sample's characteristics, the answers' scale was provided on an A4 sheet, always visible to the participants, and the questions were made verbally. The scale was translated from English to Portuguese by two experts in English-to-Portuguese translation.

### Experimental procedure

The study followed a within-person design with three conditions (Competitive, Co-active, and Collaborative). The order of the conditions was randomized using random.org. Data collection was conducted in two sessions of approximately 90 min for each pair of players. In the first session, participants signed the informed consent, were checked against exclusion criteria, and underwent motor and cognitive assessments. An occupational therapist was responsible for the assessments. Sessions were conducted by two researchers trained on the system and the assessment questionnaires. Participants were arranged in pairs (10 in total) according to their motor skill level as assessed by Section A-Shoulder/Elbow/Forearm of FMA-UE, excluding reflex activity. We limited the difference in scores between paired participants to a maximum of 10 out of 30, just considering component A without reflexes activity. The pairs were maintained for all conditions of the study.

In Session 2, we introduced the system to participants through a training phase, allowing them to play each specific game mode before the condition was tested. We ensured that they got familiar with the interface and the game by having participants playing with no time limit, just finishing when researchers considered they had understood the game's purpose and how to play it, reducing the effects of learning and novelty for each game mode. Each condition consisted of 8 consecutive rounds of 1 minute with a 5-15 seconds interval between rounds to allow participants to interact with each other, besides they were allowed to interact during the round. Between each round, the score was reset. At the end of each condition, pairs of participants answered the GEQ—Core Module and GEQ—Social Presence Module in separate rooms.

### Data analysis

Because of the ordinal nature of the measures, non-parametric statistical tests were used for data analyses. Hence, the median was used as a measure of central tendency and the interquartile range (IQR) for dispersion. To test for differences across conditions, we used Friedman's test for each of the modules' components from the GEQ, cognitive, motor, and personality impact. The Wilcoxon signed-rank test was used for pairwise comparisons, with significance values adjusted by the Bonferroni correction. Data were analyzed using IBM Statistics for Mac, Version 26.0 (Armonk, NY: IBM Corp).

To understand the impact of the level of motor function, we divided the sample into two subgroups according to the mean of FMA-UE score (42.8 ± 14.00), resulting in a group with a score higher than 42.8 (n = 12) and another below or equal to 41 (n = 8). For between-group comparisons, we used the Mann-Whitney U Test. The same method was followed to analyze the impact of the level of cognitive function and personality. Regarding the cognitive function, we divided the sample into two subgroups according to the mean of MoCA scores (21.1 ± 5.4), the group with a score higher or equal to 21 (n = 11) and below 21 (n = 9). For personality, we also divided the sample into two subgroups using the mean of extraversion component of Mini-IPIP (13.7 ± 2.9), the group with a score lower than 13 (n = 10) and higher or equal to 13 (n = 11). Finally, we analyzed the correlation between spasticity and GEQ ratings in each game mode using Pearson's correlation coefficient.

## Results

### Engagement

Concerning engagement (Table 2), in all conditions (Competitive, Co-active, Collaborative), Flow, Positive Affect, and Competence were reported as high (out of 4 points). The level of Challenge was considered low. Regarding Tension/Annoyance and Negative Affect, these were rated as very low.

**Table 2:** Medians (interquartile range) in the game experience questionnaire components—core module per condition, and Friedman's statistics across conditions

Component	Competitive	Co-active	Collaborative	Friedman's statistic	
				(Chi-Square)	*p* value
Competence	2.7 (0.8)	2.4 (1.2)	2.6 (0.8)	0.400	0.819
Sensory and Imaginative Immersion	2.3 (1.1)	2.4 (1.2)	2.3 (1.0)	0.194	0.907
Flow	3.1 (1.2)*	2.6 (1.0)*	**3.0 (1.3)**	**12.277**	**0.002**
Tension/Annoyance	0.0 (0.0)	0.0 (0.0)	0.0 (0.0)	1.727	0.422
Challenge	1.2 (1.0)*	0.6 (1.2)*	**1.2 (1.0)**	**10.959**	**0.004**
Negative Affect	0.1 (0.5)	0.0 (0.5)	0.0 (0.4)	0.167	0.920
Positive Affect	3.0 (1.1)	3.0 (0.7)	3.0 (1.0)	1.794	0.408

Bold values represent that significant differences between conditions were found. The asterisk represents significant results after pairwise comparisons

**Fig. 3:**
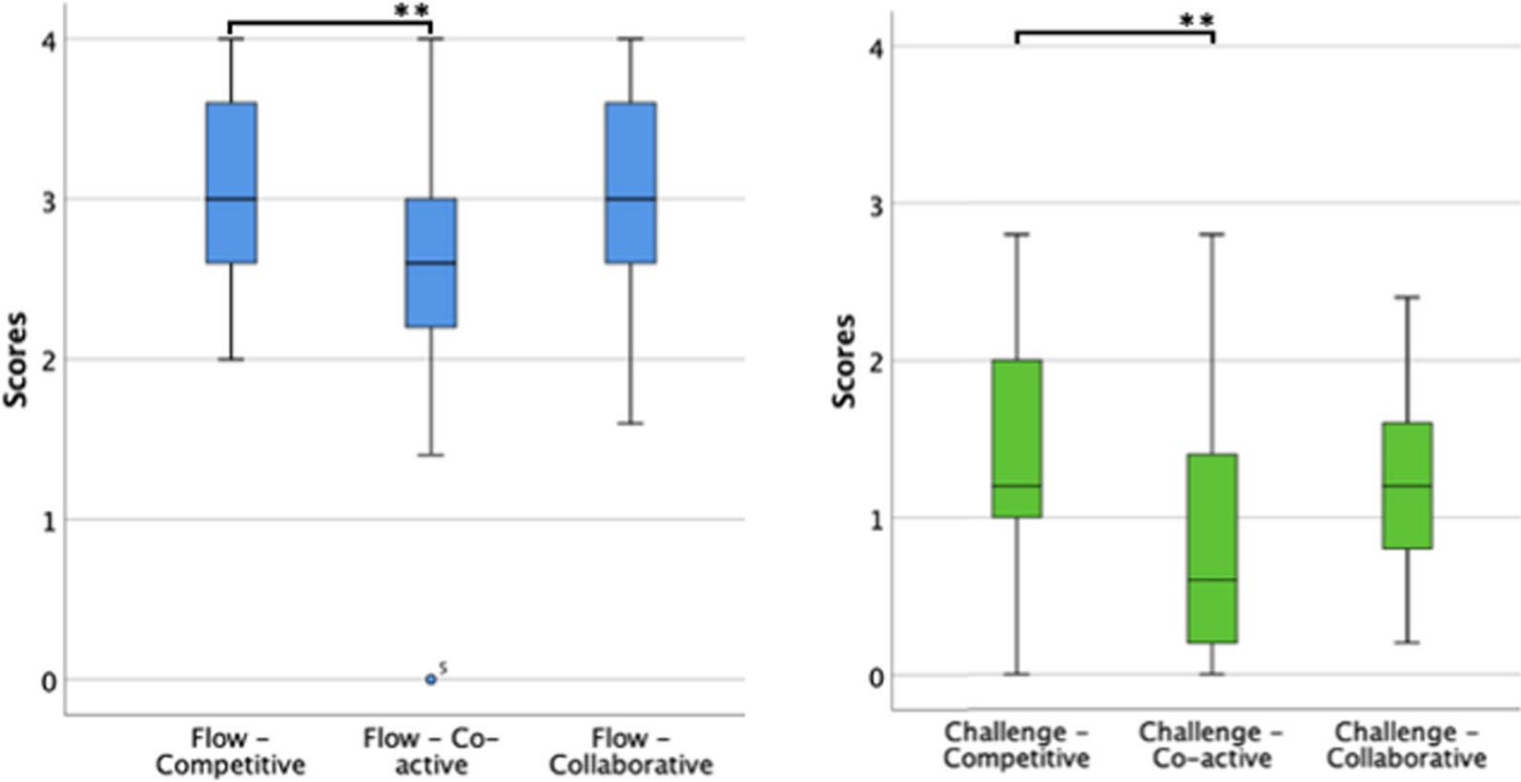
Boxplots of the components Flow and Challenge from the GEQ—Core Module per game mode. **p < 0.01.

We found significant differences across conditions in Flow (χ(2) = 12.277, p = 0.002) and Challenge (χ(2) = 10.959, p = 0.004) (Table 2, Figure 3). Pairwise comparisons revealed that Flow (Z = -2.962, p = 0.003) and Challenge (Z = 3.312, p = 0.002) were significantly higher in the Competitive mode than the Co-active mode.

### Social Involvement

**Fig. 4:**
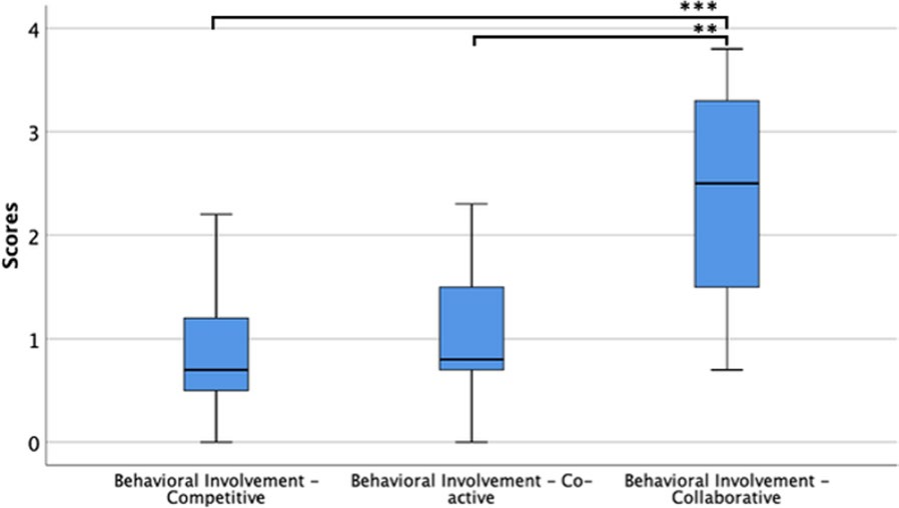
Boxplots of the component Behavioral Involvement from the GEQ—Social Presence Module per game mode, with all sample. **p < 0.01, ***p < 0.001

Regarding Social Involvement, we found significant differences across conditions in Behavioral Involvement (χ(2) = 26.694, p < 0.001) (Table 3, Figure 4). Pairwise comparisons revealed that this effect arises from the Collaborative mode being significantly higher when compared to the Competitive (Z = − 3.827, p < 0.001) and the Co-active mode (Z = − 3.684, p = 0.001). Empathy was similar and positive on the three conditions, contrasting with Negative Feelings that were low across all conditions (Table 3).

**Table 3:** Medians (interquartile range) in the game experience questionnaire components—social presence module per condition, and Friedman's statistics across conditions

Component	Competitive	Co-active	Collaborative	Friedman's statistic	
				(Chi-Square)	*p* value
Behavioral Involvement	0.7 (0.9)*	0.8 (1.1)*	2.8 (1.8)*	**26.694**	**< 0.001**
Empathy	2.3 (1.6)	2.5 (1.2)	2.6 (1.4)	2.795	0.247
Negative Feelings	0.4 (1.0)	0.6 (0.8)	0.6 (1.0)	0.689	0.709

Bold values represent that significant differences between conditions were found. The asterisk represents significant results after pairwise comparisons

### Effect of the cognitive profile

When dividing the sample into higher and lower score groups according to their MoCA score, we found that there were significant differences across conditions for both groups in Behavioral Involvement (Lower MoCA scores: χ(2) = 13.937, p < 0.001; Higher MoCA scores: χ(2) = 12.950, p = 0.002) (Table 4). Pairwise comparisons showed that both groups felt significantly more behavioral involvement with the Collaborative mode when compared to the Competitive mode (Lower MoCA scores: Z = − 2.670, p = 0.008; Higher MoCA scores: Z = − 2.803, p = 0.005) and the Co-active mode (Lower MoCA scores: Z = − 2.521, p = 0.012; Higher MoCA scores: Z = − 2.708, p = 0.007) (Table 4).

**Table 4:** Medians (Mdn) and interquartile range (IQR) for high (H MoCAs) and low (L MoCAs) Montréal cognitive assessment scores (MoCAs) for each condition (game mode), between-groups comparison (Mann–Whitney U Test), and between conditions comparison (Friedman's)

	Component	Competitive			Co-active			Collaborative			Between conditions comparison	
		*H MoCAs*	*L MoCAs*	Between-groups comp.	*H MoCAs*	*L MoCAs*	Between-groups comp.	*H MoCAs*	*L MoCAs*	Between-groups comp.	H MoCAs	L MoCAs
		*Mdn (IQR)*	*Mdn (IQR)*	*Mann-Whitney U, p-Value*	*Mdn (IQR)*	*Mdn (IQR)*	*Mann-Whitney U, p-Value*	*Mdn (IQR)*	*Mdn (IQR)*	*Mann-Whitney U, p-Value*	Chi-Square, *p-Value*	Chi-Square, *p-Value*
Core module	Competence	2.2 (0.8)	3.0 (0.8)	29.500, 0.124	**2.4 (0.6)**	**3.4 (1.2)**	**18.500, 0.017**	2.4 (0.8)	2.8 (0.5)	24.000, 0.050	1.167, 0.558	0.483, 0.786
	Sensory and Imaginative Immersion	2.3 (1.0)	2.8 (1.2)	32.000, 0.181	2.2 (1.4)	2.7 (0.9)	24.500, 0.056	2.2 (0.5)	2.8 (0.9)	29.000, 0.117	0.195, 0.907	0.581, 0.748
	Flow	**3.0 (0.8)**	3.6 (1.6)	34.500, 0.252	**2.6 (0.8)**	3.0 (1.1)	31.500, 0.170	3.0 (1.2)	3.0 (1.5)	40.500, 0.492	**9.722, 0.008**	3.379, 0.185
	Tension/Annoyance	**0.0 (0.0)**	**0.0 (0.5)**	**33.000, 0.044**	0.0 (0.0)	0.0 (0.0)	45.000, 0.366	0.0 (0.0)	0.0 (0.5)	45.500, 0.664	2.000, 0.368	3.846, 0.146
	Challenge	**1.2 (1.4)**	1.2 (1.1)	41.500, 0.541	**0.6 (1.0)**	0.6 (1.7)	45.500, 0.760	**0.8 (0.8)**	**1.5 (0.9)**	**23.500, 0.047**	**8.537, 0.014**	4.688, 0.096
	Negative Affect	**0.0 (0.3)**	**0.3 (0.5)**	**25.000, 0.045**	0.0 (0.5)	0.3 (0.5)	44.000, 0.644	0.0 (0.3)	0.0 (0.6)	49.000, 0.966	1.750, 0.417	12.643, 0.267
	Positive Affect	2.8 (0.8)	3.4 (1.2)	30.500, 0.145	2.8 (0.8)	3.0 (1.0)	32.500, 0.193	2.8 (1.4)	3.2 (1.0)	34.000, 0.235	1.514, 0.469	0.581, 0.748
Social Presence Module	Empathy	1.7 (1.3)	2.8 (0.9)	24.500, 0.057	2.3 (1.5)	2.7 (1.0)	37.500, 0.359	**2.2 (1.3)**	**2.8 (1.4)**	23.500, 0.047	2.048, 0.359	2.000, 0.368
	Negative Feelings	0.0 (0.4)	0.8 (0.9)	29.000, 0.105	0.6 (0.8)	0.4 (0.8)	45.500, 0.750	0.4 (0.6)	1.0 (1.2)	32.000, 0.180	3.879, 0.144	2.643, 0.267
	Behavioral Involvement	**0.7 (0.7)**	**1.2 (1.1)**	26.000, 0.070	**0.7 (1.2)**	**1.3 (1.4)**	38.000, 0.378	**2.3 (2.0)**	**3.0 (1.8)**	39.000, 0.423	**12.950, 0.002**	**13.937, 0.001**

Bold values represent that significant differences between conditions were found

We also found significant differences for those with higher MoCA scores in Flow (χ(2) = 9.722, p = 0.008) and Challenge (χ(2) = 8.537, p = 0.014). They considered having felt significantly less Flow (Z = 2.680, p = 0.007) and Challenge (Z = 2.499, p = 0.012) in the Co-active mode (Mdn = 2.6 (0.8)) when compared to Competitive mode.

When making a between-groups comparison, results revealed that those opponents with lower MoCA scores showed a significantly higher sense of Competence in the Co-active mode than those with higher scores (U = 18.500, p = 0.017) (Table 4). Lower MoCA scores significantly demonstrated more Tension/Annoyance and Negative Affect in the Competitive mode than those with high scores (U = 33.000, p = 0.044) and (U = 25.000, p = 0.045), respectively. Finally, the Collaborative mode was significantly more challenging for the lower MoCAs than for higher MoCAs, (U = 23.500, p = 0.047). (U = 25.000, p = 0.045), It also promoted more empathy with the lower MoCAs scores compared with, the higher MoCAs scores (U = 23.500, p = 0.047).

### Effect of motor profile and spasticity

When dividing the sample into groups according to their FMA-UE score, we found significant differences across conditions in Behavioral Involvement for both, participants with lower (χ(2) = 12.452, p = 0.002) and higher (χ(2) = 15.951, p < 0.001) FMA-UE scores. Pairwise comparison showed that both groups felt significantly more behaviorally involved with the Collaborative mode than the Competitive mode (Higher FMA-UE: Z = − 2.940, p = 0.003; Lower FMA-UE: Z = − 2.521, p = 0.012). For the lower FMA-UE group only, behavioral involvement in the Collaborative mode was also significantly higher than in the Co-active mode (Z = − 2.533, p = 0.011) (Table 5).

**Table 5:** Medians (Mdn) and interquartile range (IQR) for high Fugl-Meyer assessment-upper extremity (H FMA-UE) and low Fugl-Meyer assessment-upper extremity (L FMA-UE) for each condition (game mode), and between conditions comparison (Friedman's)

	Component	Competitive			Co-active			Collaborative			Between conditions comparison	
		*H* *FMA-UE*	*L* *FMA-UE*	Between-groups comp. *Mann-Whitney U, p-Value*	*H* *FMA-UE*	*L* *FMA-UE*	Between-groups comp. *Mann-Whitney U, p-Value*	*H* *FMA-UE*	*L* *FMA-UE*	Between-groups comp. *Mann-Whitney U, p-Value*	*H* *FMA-UE*	*L* *FMA-UE*
		*Mdn (IQR)*	*Mdn (IQR)*		*Mdn (IQR)*	*Mdn (IQR)*		*Mdn (IQR)*	*Mdn (IQR)*		Chi-Square, *p-Value*	Chi-Square, *p-Value*
Core module	Competence	2.9 (0.7)	2.2 (0.9)	23.500, 0.056	2.5 (1.0)	2.3 (1.8)	37.500, 0.413	2.8 (0.6)	2.4 (0.7)	27.500, 0.110	1.500, 0.472	0.272, 0.871
	Sensory and Imaginative Immersion	2.4 (1.2)	2.3 (1.0)	42.500, 0.669	2.6 (0.8)	2.1 (1.4)	32.500, 0.229	2.3 (0.9)	2.4 (1.1)	44.00, 0.756	0.409, 0.815	0.286, 0.867
	Flow	**3.3 (0.8)**	2.7 (1.6)	35.00, 0.314	**2.9 (1.2)**	**2.4 (1.1)**	**22.000, 0.044**	**3.1 (1.0)**	2.8 (1.6)	35.500, 0.332	**7.167, 0.028**	5.793, 0.055
	Tension/Annoyance	0.0 (0.0)	0.0 (0.3)	41.000, 0.385	0.0 (0.0)	0.0 (0.0)	42.00, 0.221	0.0 (0.6)	0.0 (0.0)	32.000, 0.077	4.769, 0.092	2.000, 0.368
	Challenge	**1.4 (1.8)**	1.1 (0.6)	40.000, 0.534	**0.6 (1.2)**	0.8 (1.0)	42.000, 0.641	**1.2 (1.3)**	1.1 (0.8)	43.000, 0.698	**6.186, 0.045**	4.867, 0.088
	Negative Affect	0.0 (0.4)	0.4 (0.7)	34.000, 0.245	0.0 (0.5)	0.4 (0.7)	34.00, 0.233	0.0 (0.4)	0.0 (0.5)	45.500, 0.827	0.963, 0.618	2,571, 0.276
	Positive Affect	3.0 (1.2)	3.0 (0.8)	43.000, 0.697	3.0 (0.7)	2.9 (0.9)	39.000, 0.484	3.0 (1.3)	3.0 (1.4)	40.500, 0.560	0.650, 0.723	1.357, 0.507
Social Presence Module	Empathy	2.4 (0.9)	1.9 (1.5)	41.500, 0.615	2.5 (1.1)	2.5 (1.8)	35.000, 0.313	2.8 (0.9)	2.4 (1.2)	36.000, 0.351	3.872, 0.144	0.194, 0.908
	Negative Feelings	0.4 (1.0)	0.1 (0.7)	34.000, 0.262	0.5 (1.1)	0.7 (0.8)	46.000, 0.871	0.5 (1.1)	0.7 (0.9)	35.500, 0.613	0.250, 0.882	0.897, 0.639
	Behavioral Involvement	**1.0 (1.6)**	**0.7 (0.2)**	35.500, 0.327	**1.4 (1.3)**	**0.6 (0.8)**	**17.000, 0.016**	**2.8 (1.5)**	**2.7 (1.8)**	46.500, 0.907	**15.951, < 0.001**	**12.452, 0.002**

Bold values represent that significant differences between conditions were found

Regarding the participants with higher FMA-UE scores, we found significant effects for Flow (χ(2) = 7.167, p = 0.028) and Challenge (χ(2) = 6.186, p = 0.045) (Table 5). Pairwise comparisons revealed that higher FMA-UE scores report significantly more Flow (Z = − 2.546, p = 0.011) and Challenge (Z = − 2.527, p = 0.012) with the Competitive mode than the Co-active mode.

When making a between-groups comparison, results show that in the Co-active mode, lower FMA-UE scores are associated with a lower sense of Flow (U = 22.000, p = 0.044) and significantly less behaviorally involved (U = 17.000, p = 0.016) when comparing with high FMA-UE scores.

To understand the relation between spasticity and ratings in the GEQ components for each game mode, we computed bivariate correlations. We found a negative correlation with Competence in Competitive mode (r(18) = − 0.477, p = 0.033) and in Flow with Co-active mode (r(18) = − 0.529, p = 0.016) and Collaborative mode (r(18) = − 0.465, p = 0.039). At last, Tension/Annoyance was also negatively correlated with Collaborative mode (r(18) = − 0.467, p = 0.038) (Table 6).

**Table 6:** Correlation Coefficient and p-value between spasticity and all Game Experience Questionnaire components for each condition (game mode)

	Component		Competitive	Co-active	Collaborative
Core Module	Competence	r	**−** **0.477**	**−** 0.375	**−** 0.288
		*p* value	**0.033**	0.104	0.219
	Sensory and Imaginative Immersion	r	**−** 0.192	**−** 0.302	**−** 0.227
		*p* value	0.417	0.196	0.336
	Flow	r	**−** 0.392	**−** **0.529**	**−** **0.465**
		*p* value	0.087	**0.016**	**0.039**
	Tension/Annoyance	r	**−** 0.186	0.229	**−** **0.467**
		*p* value	0.431	0.331	**0.038**
	Challenge	r	**−** 0.147	0.192	**−** 0.247
		*p* value	0.537	0.418	0.295
	Negative Affect	r	0.268	0.147	**−** 0.195
		*p* value	0.254	0.538	0.409
	Positive Affect	r	**−** 0.012	**−** 0.213	0.043
		*p−* *value*	0.959	0.368	0.857
Social Presence Module	*Empathy*	*r*	**−** 0.001	**−** 0.215	**−** 0.097
		*p* value	0.998	0.363	0.684
	*Negative Feelings*	*r*	**−** 0.259	**−** 0.242	**−** 0.114
		*p* value	0.270	0.304	0.633
	*Behavioral Involvement*	*r*	**−** 0.264	**−** 0.427	**−** 0.040
		*p* value	0.260	0.061	0.867

Bold values represent that significant differences between conditions were found

### Effect of personality

After dividing the sample into groups according to their Extraversion scores, we found that there were significant differences across conditions in Behavioral Involvement for both, participants with higher (χ(2) = 11.118, p = 0.042) and lower (χ(2) = 16.000, p < 0.001) scores. Pairwise comparisons showed that groups felt significantly more behaviorally involved with the Collaborative mode when compared to the Competitive mode (More extrovert: Z = − 2.668, p = 0.008, Less extrovert: Z = − 2.807, p = 0.005). Additionally, the less extrovert group only felt significantly more behaviorally involved with the Co-active mode (Z = − 2.821, p = 0.005). We also found significant differences with the less extrovert participants in Flow (χ(2) = 10.563, p = 0.005) and Challenge (χ(2) = 7.000, p = 0.030). They considered having felt significantly less Flow (Z = − 2.524, p = 0.012) and Challenge (Z = 2.501, p = 0.012) when compared to Competitive mode (Table 7).

**Table 7:** Medians (Mdn) and interquartile range (IQR) for high extraversion and low extraversion for each condition (game mode), and between conditions comparison (Friedman's)

	Component	Competitive			Co-active			Collaborative			Between conditions comparison	
		*High* *Extraversion*	*Low* *Extraversion*	Between-groups comp. *Mann-Whitney U, p-Value*	*High* *Extraversion*	*Low* *Extraversion*	Between-groups comp. *Mann-Whitney U, p-Value*	*High* *Extraversion*	*Low* *Extraversion*	Between-groups comp. *Mann-Whitney U, p-Value*	*High* *Extraversion*	*Low* *Extraversion*
		*Mdn (IQR)*	*Mdn (IQR)*		*Mdn (IQR)*	*Mdn (IQR)*		*Mdn (IQR)*	*Mdn (IQR)*		Chi-Square, *p-Value*	Chi-Square, *p-Value*
Core module	*Competence*	3.0 (0.5)	2.2 (0.7)	29.500, 0.117	2.4 (1.1)	2.5 (1.6)	46.500, 0.789	**2.8 (0.7)**	**2.3 (1.9)**	**24.000, 0.047**	1.806, 0.405	2.294, 0.318
	*Sensory and Imaginative Immersion*	2.3 (1.4)	2.6 (0.6)	39.000, 0.402	2.4 (1.4)	2.5 (1.2)	46.000, 0.761.	2.3 (1.1)	2.4 (0.9)	47.500, 0.849	1.027, 0.598	0.914, 0.633
	*Flow*	3.0 (1.3)	**3.2 (1.0)**	48.000, 0.879	2.6 (1.5)	**2.7 (0.9)**	43.500, 0.622	2.8 (1.7)	**3.0 (1.0)**	42.500, 0.569	2.970, 0.227	**10.563, 0.005**
	*Tension/Annoyance*	0.0 (0.1)	0.0 (0.0)	45.000, 0.543	0.0 (0.0)	0.0 (0.0)	45.000, 0.317	0.0 (0.1)	0.0 (0.2)	50.000, 1.000	2.600, 0.273	0.500, 0.779
	*Challenge*	1.2 (2.0)	**1.5 (0.9)**	38.500, 0.382	0.5 (1.3)	**1.0 (1.1)**	41.000, 0.493	1.3 (1.4)	**1.1 (0.8)**	48.000, 0.879	4.171, 0.124	**7.000, 0.030**
	*Negative Affect*	0.1 (0.5)	0.1 (0.4)	46.500, 0.776	0.0 (0.5)	0.3 (0.6)	42.000, 0.504	0.0 (0.6)	0.1 (0.3)	45.500, 0.700	0.095, 0.953	0.667, 0.717
	*Positive Affect*	3.0 (1.3)	3.0 (0.9)	49.000, 0.939	3.0 (1.2)	2.9 (1.7)	45.500, 0.732	3.1 (1.5)	2.9 (1.1)	43.000, 0.594	2.438, 0.296	1.556, 0.459
Social Presence Module	*Empathy*	2.6 (2.0)	1.9 (1.5)	37.500, 0.343	2.5 (1.1)	2.5 (1.6)	37.500, 0.342	2.5 (2.2)	2.6 (1.2)	47.500, 0.849	2.513, 0.285	1.282, 0.527
	*Negative Feelings*	0.4 (1.0)	0.2 (0.9)	39.000, 0.387	0.7 (1.0)	0.5 (0.8)	44.000, 0.634	0.3 (1.0)	0.7 (0.6)	44.500, 0.675	0.452, 0.798	1.267, 0.531
	*Behavioral Involvement*	**1.0 (1.0)**	**0.7 (0.5)**	31.000, 0.145	0.9 (1.0)	**0.7 (1.4)**	43.000, 0.593	**2.4 (2.8)**	**3.0 (1.7)**	47.500, 0.849	**11.118, 0.004**	**16.000, < 0.001**

Bold values represent that significant differences between conditions were found

A between-groups analysis rendered significant differences in Competence in Collaborative mode. In this mode, the more extrovert participants revealed significantly more Competence when compared with those less extrovert.

## Discussion

Here we studied the impact of three game modes (Competitive, Co-active, and Collaborative) in engagement and social involvement during a rehabilitation game for stroke survivors. Our primary purpose was to identify the most adequate multiplayer game approach for a stroke motor rehabilitation program. In a previous study with the same purpose, we analyzed the impact of different game modes in a sample of healthy community-dwelling older adults [10], where participants interacted with the game sitting side-to-side. However, in this study, we prepared the setup to have them front-to-front to enhance the experience's social impact. Also, the interaction with the game is more straightforward, as, in this study, participants interact directly with the virtual objects using a real object on a touch-sensitive horizontal screen. In the previous research, they had to move a real object, being this movement translated into the action of a virtual object on a vertical screen [10]. For the present study, we aimed to understand how motor and cognitive impairments brought by stroke, but personality as well, modulates the experience of multi-user interaction.

### Social involvement

Results showed a significant effect of game mode on Behavioral Involvement, a component that measures the extent to which players feel their actions are dependent on their co-players' actions [33]. This dependence is positive as it can foster communication, which is essential to promote social interaction. This is particularly important because of the impact that social engagement can have on health and well-being in senior populations [34] and on high levels of adherence to therapy when a game fosters support and communication between players [35]. Moreover, in a rehabilitation context, it has been shown to contribute to both higher levels of enjoyment during interaction and an increased sense of self-efficacy [36].

Concerning the different game modes, the Collaborative game mode elicited significantly more Behavioral Involvement. This is consistent with the results of our previous study with healthy older adults [10]. Although the setup was different (we used a standard desktop computer instead of an interactive table), the Collaborative mode promoted significantly more Behavioral Involvement than Co-active and Competitive modes. In the present study, we verified the same, significantly higher levels of Behavioral Involvement with the Collaborative mode when compared to the Co-active and Competitive modes in almost all participants, except the more extrovert participants and those with fewer motor difficulties, which only felt significantly more Behavioral Involvement with the Collaborative mode when compared with the Competitive mode. This suggests that participants with those characteristics (more extrovert or fewer motor difficulties) are more receptive to get involved with players with a different profile from them (i.e., with more cognitive or/and motor deficits) with non-competitive game modes. Additionally, participants with more serious cognitive difficulties report significantly more empathy with the Collaborative mode than participants with less cognitive difficulties. This supports the previous hypothesis, which points to the Collaborative mode as preferable to promote interaction.

In the Co-active mode, participants with fewer motor difficulties felt significantly more Behavioral Involvement when compared to those with more serious motor difficulties, besides values of Behavioral Involvement being reported as low. This can be interpreted as these players being more aware of how their dominance could impact their teammate, adapting their performance to motivate and engage their teammate.

It is important and still an open research question to understand how to manipulate game conditions to balance the skill levels to enable multiplayer gaming [12]. Baur et al. also acknowledge that players can have very different skills in rehabilitation, and that poses an important challenge in multiplayer games. Our data suggest that, for individuals with fewer motor skills or an extrovert personality, the use of Collaborative or Co-active game modes is preferred to promote positive gaming experiences.

### Engagement

Data from the different components of the GEQ-Core Module assessed the impact that the game experience had on participants' engagement. Overall, and irrespective of the game mode, participants reported low feelings of Tension/Annoyance and Negative Affect and high levels of Flow, Positive Affect, and Competence. Literature suggests that patients performing exercises with a co-player they already know and have a positive relationship with maximize engagement and motivation within the activity [14]. Although our experiment participants did not know each other, our study revealed that participants felt moderate levels of empathy between them while playing the multi-user game. This result is in accordance with the results by Kort et al. that measured empathy through different social settings, such as "playing alone, with virtual others, online with unknown others, online with friends/family, and with co-players physically present (friends)" [33]. They report a value of approximately 2.1 (between 0 and 4) with co-players physically present, similar to our results with Competitive mode (2.2) and Co-active and Collaborative (2.5), being that in our study, participants did not know the partner.

Flow and Challenge were components for which we also found consistent effects. When we analyzed all the sample together, we found that participants felt significantly more Flow and Challenge with the Competitive mode than the Co-active mode. This is coherent with studies that have reported competitive mode as being more motivating [35, 37]–[40]. In fact, Flow and Challenge are important cornerstones of Flow Theory [41]. In a recent study that compared a multiplayer co-active mode (according to criteria defined by Baur et al. [12]) with a solo mode, results showed no significant differences in motivation as measured by the Intrinsic Motivation Inventory [37]. Comparing this result with what we observed on our study, the Co-active mode was also the one that produced less Flow and Challenge, which can be related to motivation to some extent, as in principle, a person feels flow and challenge at the same time only when being engaged in the task [41].

When dividing the sample into sub-groups, we found that participants with better cognitive performance, the less extrovert, and the ones with higher motor skills were the specific groups that benefit more from Competitive mode in terms of Flow and Challenge. Other studies have also observed that people with low extraversion will prefer game modes where they have to compete instead of interacting as a team [42], as personality can be considered a skill in the context of multiplayer gaming, and therefore interfere with game mode preferences [41]. Thus, less extrovert people will tend to prefer contexts that do not require articulation with other players. However, in group rehabilitation, skills are potentially different between participants, being that the Competitive mode seems to be more limited in accommodating well dissimilarities or participants with potentially low performances as not all players may experience Flow. On the opposite side, Mace et al. [22] found that participants with different abilities prefer to engage in collaborative gaming, as this mode enhanced performance proportionally to partnership's mismatch.

Concerning the impact of motor function in Flow, we found significant differences between participants with high and low FMA-UE scores in the Co-active mode. When comparing both groups, participants with higher FMA-UE scores reported higher Flow levels than participants with lower scores. This result is somehow consistent with findings by Alankus et al. [43], as impaired players may find competitive modes uncomfortable. Spasticity also seems to be a factor to be considered when choosing the game mode. Our results show that it was negatively correlated with Flow in the Co-active and the Collaborative mode. This correlation suggests that participants with more spasticity felt less Flow with multiplayer modes where they had to engage in teamwork. Overall, the Competitive mode seems to be more suitable to promote Flow, being that participants with more spasticity also reported less Competence in the Co-active mode. Indeed, competitive games have been previously reported as more motivating by people with disabilities within the context of rehabilitation, as they produce more intense performances and are associated with more movement repetition [14, 38, 40]. Interestingly, Tension/Annoyance was negatively correlated with spasticity, which was not expected, as stroke severity is related to cognitive affectation [44].

When comparing participants with higher and lower cognitive deficits, we found that participants with higher cognitive deficits felt Collaborative mode more challenging. This result is in line with what we qualitatively observed during the experiment. Participants typically took more time to understand the goal and mechanics of this game mode. In our previous study, we have also reported collaborative gaming with healthy elderly as being more cognitively demanding because participants need to coordinate strategies, which requires more attention [10]. This is particularly relevant for clinical practice, as participants with lower cognitive deficits reported to feel significantly more Competence in the Co-active mode. This higher sense of Competence can be due to the higher combined team score, as the Co-active mode allows participants to contribute disproportionately for the score, compensating for possible co-players' difficulties. Furthermore, participants with higher cognitive deficits reported significantly more Negative Affect and Tension/Annoyance in the Competitive mode, which suggests that this game mode must be used with caution within-group rehabilitation settings, particularly in multi-user settings. Still, overall ratings of negative affect were very low (0.38 out of 4).

### Limitations and future directions

This study has some limitations that should be acknowledged. For a better comparison with the state-of-the-art, it would have been useful to have added the Intrinsic Motivation Inventory (IMI) as an outcome measure, as it is widely used in this type of research [14, 15, 23, 39]. Another limitation was that we used the same rehabilitation cones as interfaces for everyone, which can have impacted differently participants with different skills. However, the cone was chosen over other objects as its manipulation can be facilitated according to patients' ability and/or preference. We also consider a limitation the impossibility to verify if previous experience with video games impacted engagement and social involvement as only 5 of the participants reported previous experience.

Regarding the data analysis, our sample size is relatively small for some of the statistical analyses performed, and results should be considered with caution. Finally, our game and its different variants were carefully designed to be as similar and generalizable as possible. However, the specific design of the game can potentially influence the generalization of results [45]. Additionally, if each game was specifically designed for each game mode's characteristics, their impact could also be different. Hence, caution assuming generalization should be taken with different game mechanics or modes of interaction than those studied here. As for future work, one possible next step is to explore other variants of game modes, such as combat or object competition, in the Competitive mode. The Cooperative mode also seems very interesting for rehabilitation settings. It allows different roles in the same game, which can be used to balance players with different skills and better fulfill participants' personal interests and motivations. At last, we consider important to understand if these results are similar in the case of the group size increases, but also to better understand the relationship between different settings with familiar and non-familiar pairs and various game modes as there is research and therapeutic interest on home-based technologies for stroke rehabilitation [46].

## Conclusions

This study indicates that the Collaborative mode seems to be the more balanced game mode as it promotes significantly more Behavioral Involvement than the Competitive and Co-active modes. Simultaneously, it is not statistically different in terms of Flow and Challenge compared with the Competitive and Co-active modes. Conversely, the Co-active mode promotes significantly less Behavioral Involvement than the other two game modes. Competitive mode elicits significantly more Flow and Challenge than the Co-active mode, being participants with better cognitive performance, the less extrovert, and the ones with higher motor skills that benefit more from it. Participants with higher cognitive deficits tend to feel more competent with the Co-active mode.

To conclude, our results suggest that collaboration is the more suitable gaming strategy to promote social involvement during a multi-user motor rehabilitation setting, with the potential of increasing adherence and the effectiveness of therapy. However, motor and cognitive ability and personality should also be considered when designing personalized tasks.

## Data Availability

The descriptive measures, Friedman's test, Mann-Whitney U Test, Pearson's correlation coefficient from which the conclusions are drawn are provided in the article. Raw data is available from the corresponding author on a reasonable request.

## Abbreviations

MoCA: Montreal Cognitive Assessment
H MoCas: Higher Montreal Cognitive Assessments (participants with a score higher than the average)
L MoCas: Lower Montreal Cognitive Assessments (participants with a score lower than the average)
H FMA-UE: Higher Fugl-Meyer Assessments (participants with a score higher than the average)
L FMA-UE: Lower Fugl-Meyer Assessments (participants with a score higher than the average)
IQR: Interquartile range
AnTS: Analysis and tracking system
Mini-IPIP: Short version of International Personality Item Pool
GEQ: Game Experience Questionnaire
Mdn: Median
